# Mesenchymal stromal cell‐derived factors promote the colonization of collagen 3D scaffolds with human skin cells

**DOI:** 10.1111/jcmm.15507

**Published:** 2020-07-14

**Authors:** Raluca Tutuianu, Ana‐Maria Rosca, Madalina Georgiana Albu Kaya, Vasile Pruna, Tiberiu Paul Neagu, Ioan Lascar, Maya Simionescu, Irina Titorencu

**Affiliations:** ^1^ Institute of Cellular Biology and Pathology “Nicolae Simionescu” of the Romanian Academy Bucharest Romania; ^2^ INCDTP—Division Leather and Footwear Research Institute Bucharest Romania; ^3^ University of Medicine and Pharmacy “Carol Davila” Bucharest Romania

**Keywords:** collagen scaffold, mesenchymal stromal cells conditioned medium, regenerative medicine, skin wound healing

## Abstract

The development of stem cell technology in combination with advances in biomaterials has opened new ways of producing engineered tissue substitutes. In this study, we investigated whether the therapeutic potential of an acellular porous scaffold made of type I collagen can be improved by the addition of a powerful trophic agent in the form of mesenchymal stromal cells conditioned medium (MSC‐CM) in order to be used as an acellular scaffold for skin wound healing treatment. Our experiments showed that MSC‐CM sustained the adherence of keratinocytes and fibroblasts as well as the proliferation of keratinocytes. Moreover, MSC‐CM had chemoattractant properties for keratinocytes and endothelial cells, attributable to the content of trophic and pro‐angiogenic factors. Also, for the dermal fibroblasts cultured on collagen scaffold in the presence of MSC‐CM versus serum control, the ratio between collagen III and I mRNAs increased by 2‐fold. Furthermore, the gene expression for α‐smooth muscle actin, tissue inhibitor of metalloproteinase‐1 and 2 and matrix metalloproteinase‐14 was significantly increased by approximately 2‐fold. In conclusion, factors existing in MSC‐CM improve the colonization of collagen 3D scaffolds, by sustaining the adherence and proliferation of keratinocytes and by inducing a pro‐healing phenotype in fibroblasts.

## 1 INTRODUCTION

The high demand of skin substitutes for clinical treatment and the low availability of autografts and allografts increased the interest in tissue engineering for skin therapy.^1^ Although the skin has the capacity of self‐healing, when a wide‐range damage occurs (pressure ulcers or burns) or subsequent to an illness (diabetes, ischaemia, cancer or AIDS),^2^ the wound repair decreases significantly. The consequence of such pathology, which affects elderly people, in particular, is a poor quality of life, disability, increased pain, depression and social isolation.^3^ A major problem in the treatment of these patients is the high rate of reoccurrence and the resistance to conventional treatment.^4^

There are two main research directions for the treatment of chronic wounds: innovative biomaterials and the use of stem cells, each having their own advantages. The skin comprises cells and extracellular matrix, with the latter providing mechanical strength and modulating cell proliferation and differentiation.^5^ When the tissue damage is extensive, the sole delivery of cells without a matrix results in poor survival and engraftment.^6^ Therefore, in order to improve tissue regeneration, the use of biomaterials is particularly useful, with collagen being the most explored, due to its high biocompatibility, biodegradability, weak antigenicity,^7^ availability and possibility to be processed into porous structures, which provide a suitable environment for cell growth and proliferation and facilitates the supply of nutrients and oxygen.^8^, ^9^

On the other hand, cellular therapy for wound healing using mesenchymal stromal/stem cells (MSC) is based on the differentiation potential, bioavailability, low immunogenicity and, most importantly, on the secretion of numerous soluble factors promoting cell migration, angiogenesis and granulation tissue formation by this type of cells.^10^ However, major drawbacks in the use of cellular therapy are the time‐consuming process of in vitro expansion and also the donor's variability associated with an uncertain therapeutic outcome.^11^ To avoid these inconveniences, MSC secretome in the form of conditioned medium (CM) could be standardized for large scale use as an ‘off the shelf product’.^12^, ^13^, ^14^

In this context, we aimed to combine these two approaches by supplementing a type I collagen scaffold with MSC‐CM, making use of the new concept of ‘cell‐free therapy’, in order to obtain an improved biomaterial, with boosted therapeutic properties. Thus, in this paper, we provide evidence that factors within the MSC‐CM support the colonization of a three‐dimensional (3D) type I collagen scaffold with the main types of skin cells (keratinocytes and fibroblasts) and they have the capacity to attract epithelial and endothelial cells, which is important for enhancing the re‐epithelialization and angiogenesis processes. Moreover, the presence of MSC‐released factors induces a pro‐healing phenotype in fibroblasts, enabling their contribution to wound contraction, granulation tissue formation and scaffold biodegradation.

## 2 MATERIALS AND METHODS

### 2.1 Cell culture techniques

All the procedures were approved by the Institutional Ethical Committee (180/27.09.2018), in accordance to the most recent version of the Helsinki declaration of World Medical Association (Ethical Principles for Medical Research Involving Human Subjects, October 2008). *Human bone marrow‐derived MSC* were isolated using a modified protocol established by our group.^15^ Briefly, MSC seeded at 10^5^/cm^2^ were grown in DMEM 4.5 g/L glucose supplemented with 15% foetal bovine serum (FBS), 1% non‐essential amino acids, 300 UI/mL penicillin, 300 mg/mL streptomycin and 150 mg/mL neomycin, at 37°C and 5% CO_2_. The cells were characterized following the indications of the International Society for Cellular Transplantation.^16^

The multilineage potential of bone marrow‐isolated MSCs was tested by inducing their differentiation into adipogenic, osteogenic and chondrogenic lineages. *Adipogenic differentiation:* pre‐confluent cells were incubated for 3 weeks in adipogenic differentiation medium (DMEM with 10% FBS, 10^−6^ M dexamethasone, 100 μM indomethacin and 1% insulin transferrin selenite supplement—ITS, Sigma‐Aldrich). The lipid accumulation was determined by Oil Red O (Sigma‐Aldrich) staining. *Osteogenic differentiation*: confluent cells were incubated in osteogenic differentiation medium (DMEM with 10% FBS, 10^−7^ M dexamethasone, 10 mM β‐glycerophosphate and 0.3 mM ascorbic acid). After 3 weeks of incubation, the cells were exposed to von Kossa stain to highlight calcium deposits. *Chondrogenic differentiation*: cells were detached with trypsin and 2.5 × 10^5^ cells were added to 15‐ml Falcon tubes containing 500 μl chondrogenic differentiation medium (high glucose‐DMEM with 10 ng/ml TGF‐β3, 10^−7^ M dexamethasone, 50 μg/ml ascorbate‐phosphate, 40 μg/ml proline, 100 μg/ml pyruvate, 50 mg/ml ITS—Sigma‐Aldrich). After centrifugation, the pellets were maintained at 37°C for three weeks. Next, the pellets were embedded in paraffin and 5 μm thick sections were obtained using a Leica microtome. The sections were dehydrated and stained with Alcian blue (Sigma‐Aldrich) to highlight the acid mucopolysaccharides.

For CM collection, pre‐confluent MSC from passage 4‐7 were washed with PBS and incubated with DMEM supplemented with 1% non‐essential amino acids for 24 h. After that, CM was collected and centrifuged at 2000 g for 25 minutes, and the supernatant stored at −80°C.

*Primary dermal fibroblasts* (DF) were isolated from adult skin samples (normal mammary samples from plastic surgery procedures, with informed consent) using the explant technique, in DMEM 4.5 g/L glucose supplemented with 15% foetal bovine serum (FBS), 1% non‐essential amino acids, 300 UI/ mL penicillin, 300 mg/mL streptomycin and 150 mg/mL neomycin, seeded at ~10^5^ cells/cm^2^ and used at passages 4‐10.

The human keratinocyte cell line HaCaT (CLS GmbH) and the human endothelial cell line EA.hy926 (ATCC^®^ CRL‐2922^™^) were used according to the manufacturer's instructions and cultured in DMEM 4.5 g/L glucose supplemented with 10% foetal bovine serum (FBS), 1% non‐essential amino acids, 300 UI/ mL penicillin, 300 mg/mL streptomycin and 150 mg/mL neomycin.

### 2.2 Flow cytometry

MSCs were detached with accutase (Sigma‐Aldrich) and exposed to the antibodies that fulfil the minimal criteria for the definition of human MSC (FITC coupled antibodies for negative markers—CD45, CD34—and PE coupled antibodies for the positive markers—CD44, CD73, CD90, CD105, all from BD Biosciences) according to the manufacturer's protocol. The samples containing 10^5^ cells were analysed using a Beckman Coulter 3 laser Gallios cytometer and the data were analysed with Summit software v4.3 (Cytomation, Inc).

### 2.3 Characterization of human dermal fibroblasts by immunocytochemistry

Human dermal fibroblasts cultured on glass coverslips were fixed and permeabilized in 4% PFA with 0.1% Triton X and blocked with 1% BSA, before staining with mouse anti‐vimentin (Sigma‐Aldrich), mouse anti‐fibronectin (Sigma‐Aldrich) and rabbit anti‐collagen type I (Abcam) for 1 h at 37°C. After washing the primary antibody, the cells were incubated with secondary antibodies conjugated with: rabbit Alexa 488 or mouse Alexa 568 (Thermo Scientific). The coverslips were mounted with Fluoroshield with DAPI (Thermo Scientific) and visualized using a Zeiss Observer D1 microscope.

### 2.4 Collagen scaffold preparation

The type I fibrillar collagen gel was extracted from calf hide by acid and alkaline treatments, Briefly, the gels containing 1.2% collagen and having a pH of 7.4 were cross linked with 0.5% glutaraldehyde (Merck, Germany), followed by freeze‐drying at −40°C for 4 hours, using a Delta 2‐24 LSC Christ (Germany) as previously described.^17^, ^18^ The spongious forms were characterized by scanning electron microscopy using a QUANTA INSPECT F SEM device equipped with a field emission gun (FEG) with a resolution of 1.2 nm, as previously described 17. For in vitro testing, the scaffolds were sterilized in 70% ethanol overnight, followed by several rinses in sterile PBS and maintained in DMEM without serum for at least 12 hours.

### 2.5 Assessment of collagen scaffold capacity to support colonization of human keratinocytes and dermal fibroblasts

Keratinocytes and dermal fibroblasts were seeded on collagen scaffolds (3 × 10^5^ and 2 × 10^5^, respectively) and incubated in complete medium at 37°C and 5% CO_2_ atmosphere. The scaffolds were washed with PBS, fixed in 4% PFA, embedded in Shandon Cryomatrix (Thermo Scientific) and cryosectioned using a Leica cryotome in order to obtain 4 μm thick slices. The slices were subsequently subjected to haematoxylin‐eosin, DAPI, eosin‐Hoechst or Ayoub‐Shklar staining. For the first staining, the slices were incubated for 7 minutes with haematoxylin and 5 minutes with eosin Y, mounted in glycerol and visualized using a Zeiss Observer D1 microscope. For eosin‐Hoechst staining, after incubation (2 min) in eosin Y, the slices were differentiated with 70% ethanol, washed with distilled water, stained (10 min) with Hoechst (1 mg/ml), washed, mounted and visualized. For Ayoub‐Shklar staining, the slices were incubated with acid fuchsin 5% for 5 min, followed by 45 min in aniline blue‐orange G solution, washed and mounted. Additionally, keratinocytes and fibroblasts were stained with CellTracker^™^ Red CMTPX Dye (Thermo Fisher Scientific), seeded on scaffolds and kept at 37°C and 5% CO_2_ atmosphere. After 3 days, the scaffolds were washed with PBS and visualized by IVIS Spectrum CT System (Perkin Elmer, Caliper, LifeSciences).

### 2.6 Qualitative assessment of the viability of keratinocytes and fibroblasts cultured on 3D collagen scaffolds

The collagen scaffolds cultured with keratinocytes and fibroblasts for 5 days were stained with LIVE/DEAD^™^ Viability/Cytotoxicity Kit (Thermo Fisher Scientific). Briefly, the scaffolds were washed with warm PBS and incubated for 30 minutes in a mixture of 2 μM calcein‐AM and 4 μM ethidium homodimer‐1. Next, the scaffolds were fixed in 4% PFA, embedded in Shandon Cryomatrix (Thermo Scientific) and cryosectioned. The 4 μm thick slices obtained were subsequently visualized using a Zeiss Observer D1 fluorescence microscope after being mounted with Fluoroshield with DAPI (Thermo Scientific).

### 2.7 Evaluation of the effect of MSC‐CM on the adherence and proliferation of keratinocytes and fibroblasts in 2D culture system and 3D collagen scaffolds

The assessment of adherence was performed by seeding keratinocytes and fibroblasts in complete medium, serum‐free DMEM and MSC‐CM as follows: (i) 5 × 10^4^ keratinocytes and 2.5 × 10^4^ fibroblasts/well in a 96‐well plate and (ii) 10^5^ cells/scaffold tailored for 96‐well plate, in triplicate for each condition. The cells were incubated for 24 h followed by XTT test (Molecular Probes) according to the manufacturer's instruction. The results were presented as percentage of the positive control (serum supplemented). The dynamics of keratinocytes adherence in 2D culture system was assessed using the xCELLigence RTCA (Acea Biosciences, Inc), which measures electrical impedance in real time, using E‐plates 16 coated with high‐density gold arrays and translates it in cell index values, was used. Thus, 5 × 10^4^ keratinocytes/well were seeded in quadruplicates on an E‐plate in corresponding media (MSC‐CM versus serum and no‐serum controls) and the cellular index was measured in real time for 24 hours.

For proliferation assessment, keratinocytes and fibroblasts were seeded in complete medium as follows: (i) 10^4^ and 3 × 10^3^ cells/well in 96‐well plates, respectively and (ii) 10^5^ cells/well from each cell type/scaffold tailored for 96‐well plate, in triplicate for each condition. After 24 h, the cells/scaffolds were washed with PBS and incubated in the corresponding media (ie MSC‐CM, serum and no‐serum media). After 5 days, for both 2D and 3D settings, the wells/scaffolds were washed with PBS. Also, the scaffolds were moved to new wells in order to evaluate only the cells within the scaffolds. The proliferation was evaluated by XTT assay (Molecular Probes) and the results were given as percentage of the cells incubated in serum condition (positive control).

### 2.8 In vitro wound healing assay (scratch test)

Keratinocytes, fibroblasts and endothelial cells were grown to confluence in 96‐well. Briefly, the monolayer was scratched transversely using a 200‐μl pipette tip, washed with PBS and incubated in complete, MSC‐CM and serum‐free media. The cells were photographed immediately after the addition of the media‐ 0 h and at 8 h for endothelial cells and 14 h for keratinocytes and fibroblasts. The migration of the cells was quantified by measuring the area covered by cells using ImageJ software (NIH).

### 2.9 Chemotaxis assay to evaluate the chemotactic effect of MSC‐CM

The chemotactic properties of MSC‐CM were evaluated using the xCELLigence RTCA system (Acea Biosciences, Inc). Briefly, cell suspensions (10^5^ cells/100 μl medium) of each cell type (keratinocytes, fibroblasts, endothelial cells) were added onto the upper well of the CIM‐plate (a modified Boyden chamber, made by a polyethylene terepthalate membrane with 8‐μm pores and microelectrodes onto their inner face that generate impedance signal upon contact with cells). The tested media (completed medium, CM and serum‐free medium) were added to the lower well of the CIM‐plate and the cell migration through the membrane was monitored in real time for up to 24 h.

### 2.10 Real‐time PCR

Total RNA was extracted from fibroblasts after 5 days in culture using the TRIzol reagent (Thermo Fisher Scientific) and cDNA was synthesized starting from 1 μg of total RNA employing SENSIFAST cDNA Synthesis Kit (Bioline). Real‐time PCR was performed using The SensiFAST^™^ SYBR Hi‐ROX Kit (Bioline) optimized amplification conditions. The experiments were performed three times in triplicate for each gene. The primer sequences are given in the Supporting Information (Table S1). The analysis was done using the comparative CT method and β‐actin was employed for internal normalization.

### 2.11 Assessment of the cytokine profile of MSC‐CM

The cytokine profile was analysed using Human Angiogenesis Array (R&D Systems). Briefly, MSC‐CM was mixed with the biotinylated detection antibody cocktail and incubated with nitrocellulose membranes. The washed membranes were incubated with Streptavidin—HRP. Cytokine detection was performed using a FUJIFILM Luminescent Image analyser LAS‐3000. The pixel density was quantified by TotalLab Quant software.

### 2.12 Statistical analysis

Data were analysed with GraphPad Prism 5.0 (GraphPad Software, Inc) and presented as mean ± SD of three independent experiments, unless otherwise stated. Comparison of multiple groups was done by ANOVA. Two‐group analysis was carried out by Student t test. Probability values (*P*) < .05 were considered significant (*, *P* < .05, **, *P* < .01, ***, *P* < .001, ns, not significant).

## 3 RESULTS

### 3.1 Characterization of human bone marrow‐derived MSCs and dermal fibroblasts

**FIGURE 1:**
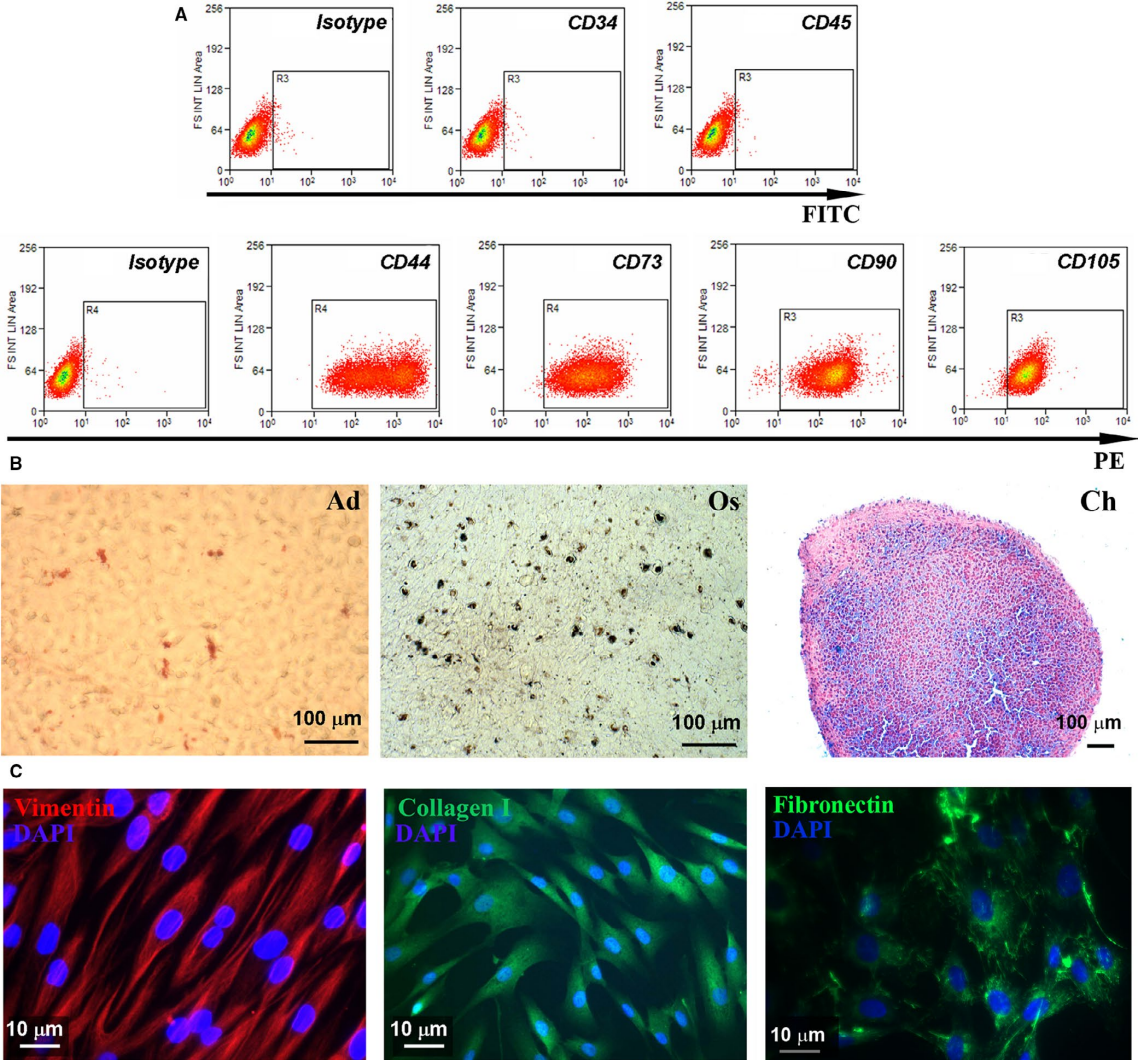
Characterization of human MSC and dermal fibroblasts. A, Expression of specific surface markers for MSC as determined by flow cytometry assay. Note that the cells are negative for the haematopoietic markers CD34 and CD45 and over 95% positive for CD44, CD73, CD90 and CD105. B, Analysis of the multipotent capacity of MSC by their ability to generate adipocytes (left, Oil Red O staining), osteocytes (middle, von Kossa staining) and chondrocytes (right, Alcian blue staining) when cultured in specific conditions. C, Immunocytochemistry images indicating the presence of vimentin (left), collagen I (centre) and fibronectin (right) in human dermal fibroblasts

MSCs isolated from human bone marrow aspirate were characterized following the guidelines of the International Society for Cellular Transplantation. As shown by the flow cytometry analysis (Figure 1A), the cells did not exhibit the presence of the haematopoietic markers CD34 and CD45 (both under 1%) and they were positive for CD44, CD73, CD90 and CD105 in a proportion of more than 95% each. As depicted in Figure 1B, when incubated for three weeks in the specific inductive conditions, the cells were able to differentiate towards the aforementioned lineages. Thus, the cells accumulated lipid droplets in their cytoplasm (as shown by Oil Red O staining) that is indicative of adipogenic differentiation. The osteogenic induction was revealed by staining the calcium deposits in the extracellular matrix with von Kossa method, while the Alcian blue staining of acid mucopolisaccharids confirmed the chondrogenic differentiation.

In order to characterize the dermal fibroblasts isolated by the explant method from human skin samples, the expression of characteristic fibroblast markers: vimentin, collagen I and fibronectin, was assessed by immunocytochemistry. As shown in Figure 1C, all these proteins were present in the isolated cells.

### 3.2 The 3D collagen scaffold allows the colonization with human skin cells

**FIGURE 2:**
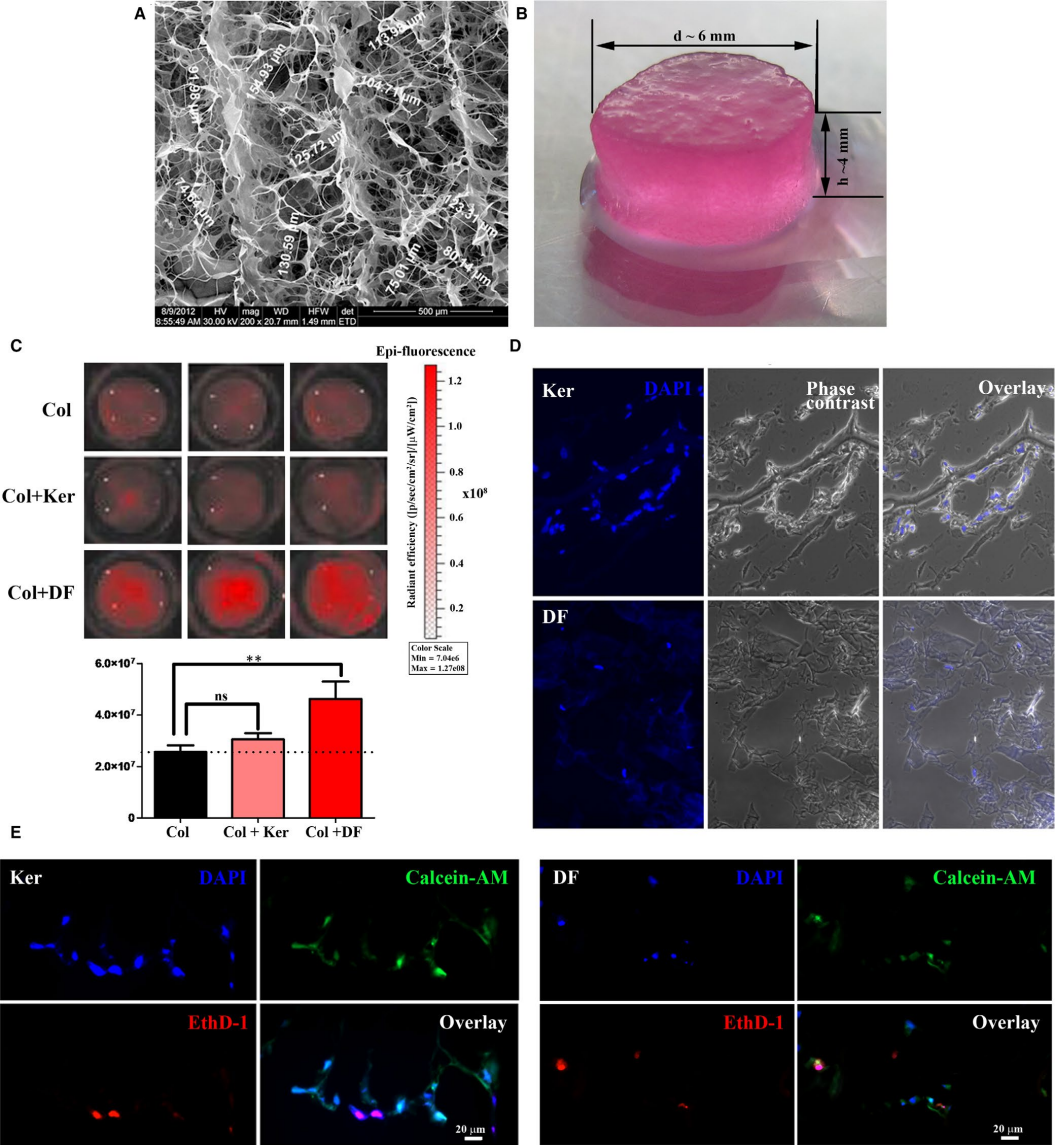
Colonization of type I collagen scaffold with skin cells: A, Electron scanning microscopy image showing the three‐dimensional aspect of the type I collagen scaffold; B, Image showing the aspect and dimensions of a scaffold pre‐incubated in DMEM, tailored for 96 well plates, prior to cell seeding; C, Collagen scaffolds (Col) after 72 h of culture with fluorescently labelled keratinocytes (Col + Ker) and fibroblasts (Col + DF); the diagram below shows the quantification of the fluorescent signal; D, DAPI nuclear staining showing the colonization of collagen scaffolds with keratinocytes (upper panel) and dermal fibroblasts (lower panel) after five days in culture; E, viability assessment of keratinocytes (left panel) and dermal fibroblasts (right panel) grown in collagen scaffolds for 5 days. The viable cells are stained with calcein‐AM (green) and the nuclei of dead cells are labelled with EthD‐1 (red)

Scanning electron microscopy revealed a three‐dimensional structure with interconnected pores, having diameters between 75 and 150 μm (Figure 2A). The macroscopic aspect of 96‐wells plate tailored scaffold, pre‐conditioned by incubation in DMEM for 24 hours prior to cell culture, with dimensions of ~4 mm in height and ~6 mm in diameter, is shown in Figure 2B. In order to establish the capacity of keratinocytes and dermal fibroblast to grow and survive on this scaffold, the CMTPX labelled cells were seeded on the pre‐conditioned sponges. The collagen scaffold itself is auto‐fluorescent, making difficult to reveal the presence of keratinocytes; the overall fluorescence was not statistically significant in comparison to the collagen scaffold alone (Figure 2C). However, the presence of fibroblasts was easily detectable, as the fluorescence intensity of the collagen seeded with labelled fibroblasts compared to bare collagen scaffold controls was twice as high and statistically relevant.

After 5 days in culture, both cell types were found inside the 3D collagen structure as shown by the presence of stained nuclei with DAPI on cryosections (Figure 2D), supporting the capacity of the scaffold to be colonized.

Next, the cell viability was assessed using a live/dead cytotoxicity kit and the presence of viable cells is evident after 5 days of culture on collagen scaffolds with some rare dead cells indicated by the red‐stained nuclei, positive for ethidium homodimer 1—EthD‐1 (Figure 2E).

### 3.3 MSC‐CM impacts the properties of keratinocytes and dermal fibroblasts in 2D classical culture system

Before testing the influence of MSC‐CM on the colonization of the collagen scaffold with skin cells, we assessed the capacity of CM to support keratinocytes and dermal fibroblasts adherence, proliferation, migration and chemoattraction in classical 2D culture system. Thus, MSC‐CM sustained the adherence of keratinocytes similarly (96.5 ± 4.5%) to the positive control (100% adherence), as shown in Figure S1. Knowing that keratinocytes are slow adherent cells, we examined the impact of MSC‐CM on the adherence process in real time using the xCELLigence system. The results showed that MSC‐CM was able to induce keratinocyte attachment faster than the serum supplement (Figure S1). At 2 h post‐seeding, the cellular index was significantly higher for keratinocytes incubated in MSC‐CM (1.4 ± 0.4) in comparison to the positive control (0.6 ± 0.03).

Also, the proliferation of keratinocytes incubated in MSC‐CM was similar to the positive control (90 ± 14% versus 100% in serum control) and significantly higher than the negative control (45 ± 14.2%), as illustrated in Figure S1.

**FIGURE 3:**
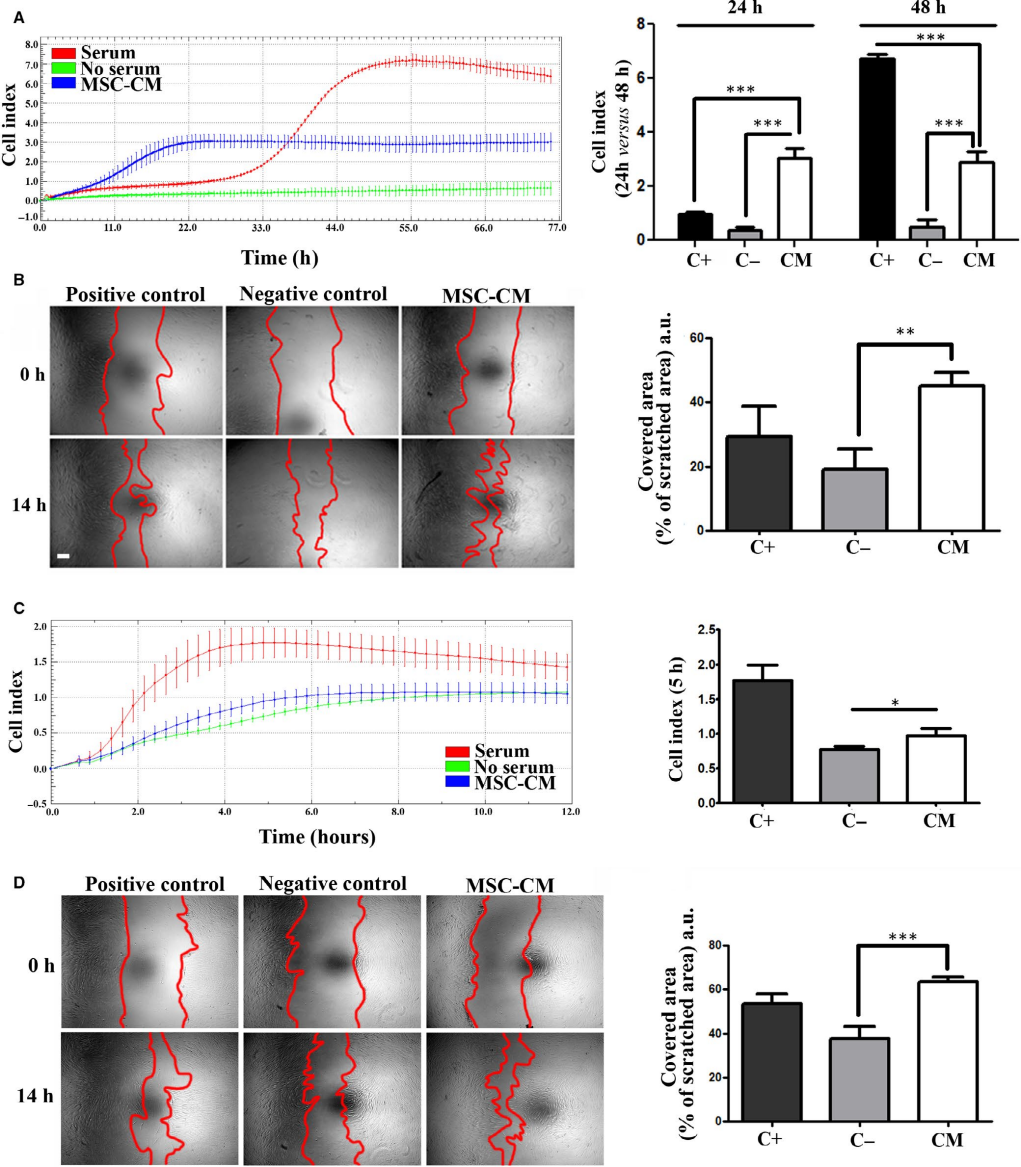
Assessment of the chemoattractive and motility stimulating effects of MSC‐CM on keratinocytes and dermal fibroblasts: A, Time‐dependent chemotactic migration of keratinocytes. The diagram on the right illustrates the keratinocyte migration index at 24 h versus 48 h, depicting the higher chemotactic effect of MSC‐CM at earlier time compared to serum. B, Keratinocytes migration assayed by the Scratch test: (left) phase‐contrast microscopy showing the scratched area at t = 0 h and 14 h later and (right) the quantification of covered area as percentage of the initial scratched area. C, Time‐dependent assessment of chemotactic migration of dermal fibroblasts in response to CM versus serum and no‐serum control. The diagram on the right illustrates the migration index at 5 h. D, Scratch test assay on fibroblasts in the presence of MSC‐CM versus positive (serum) or negative (serum‐free) controls media: (left) phase‐contrast microscopy showing the scratched area at t = 0 h and 14 h later and (right) the quantification of covered area as percentage of the initial scratched area

Next, we evaluated the MSC‐CM chemoattractant effect for keratinocytes using the xCELLigence system. The real‐time reading of cellular index indicated a strong attraction exerted by the MSC‐CM (Figure 3A). Interestingly, the dynamics of the keratinocyte migration towards the chemoattractant stimuli was different for MSC‐CM in comparison to the serum. Thus, the cellular index measured at 24 h was about 3 times higher for MSC‐CM in comparison to serum (3 ± 0.3 versus 0.95 ± 0.1 respectively), suggesting a significantly higher chemoattractant effect of the secretome. Moreover, the scratch assay showed that MSC‐CM had a strong impact on keratinocytes migration as well (Figure 3B). Thus, MSC‐CM induced a coverage of 45 ± 4% of the scratched area in comparison to 29 ± 9.7% in EGF treated cells (positive control) and 19 ± 6.5% in negative control. Altogether, these data are indicative that MSC‐CM can support the re‐epithelialization process.

Regarding the effect of MSC‐CM on the adherence, proliferation, migration and chemoattraction of fibroblasts in 2D culture system, the results indicated that, although the values were not statistically significant, MSC‐CM promoted the adherence of fibroblasts to the culture surface, as the percentage of MSC‐CM treated cells were higher than the negative control: 95 ± 19.8% versus 77 ± 15% (Figure S1). However, the proliferation assay performed after 5 days in culture in the presence of MSC‐CM clearly indicated the lack of impact of MSC‐released factors on fibroblast proliferation: 40 ± 6% for cells incubated in MSC‐CM versus 40 ± 2% in the no‐serum control (Figure S1).

Also, the data obtained by xCELLIgence assay indicated that MSC‐CM had only a mild chemoattractive effect on dermal fibroblasts (Figure 3C). Thus, at 4 h, the cellular index measured for the serum control was maximum, while the index of MSC‐CM sample was 2 times reduced versus the positive control, close to that for the negative control (the difference between the MSC‐CM sample and the negative control was not statistically relevant after 6 h). However, MSC secretome had a strong impact on fibroblast motility, since after 14 h of incubation in MSC‐CM, cells covered 64 ± 2% of wounded area, compared to 54 ± 4% for positive serum control and 37 ± 5.7 for negative control, as revealed by the scratch tests (Figure 3D).

### 3.4 MSC‐CM promotes the colonization of collagen scaffolds with keratinocytes and fibroblasts

**FIGURE 4:**
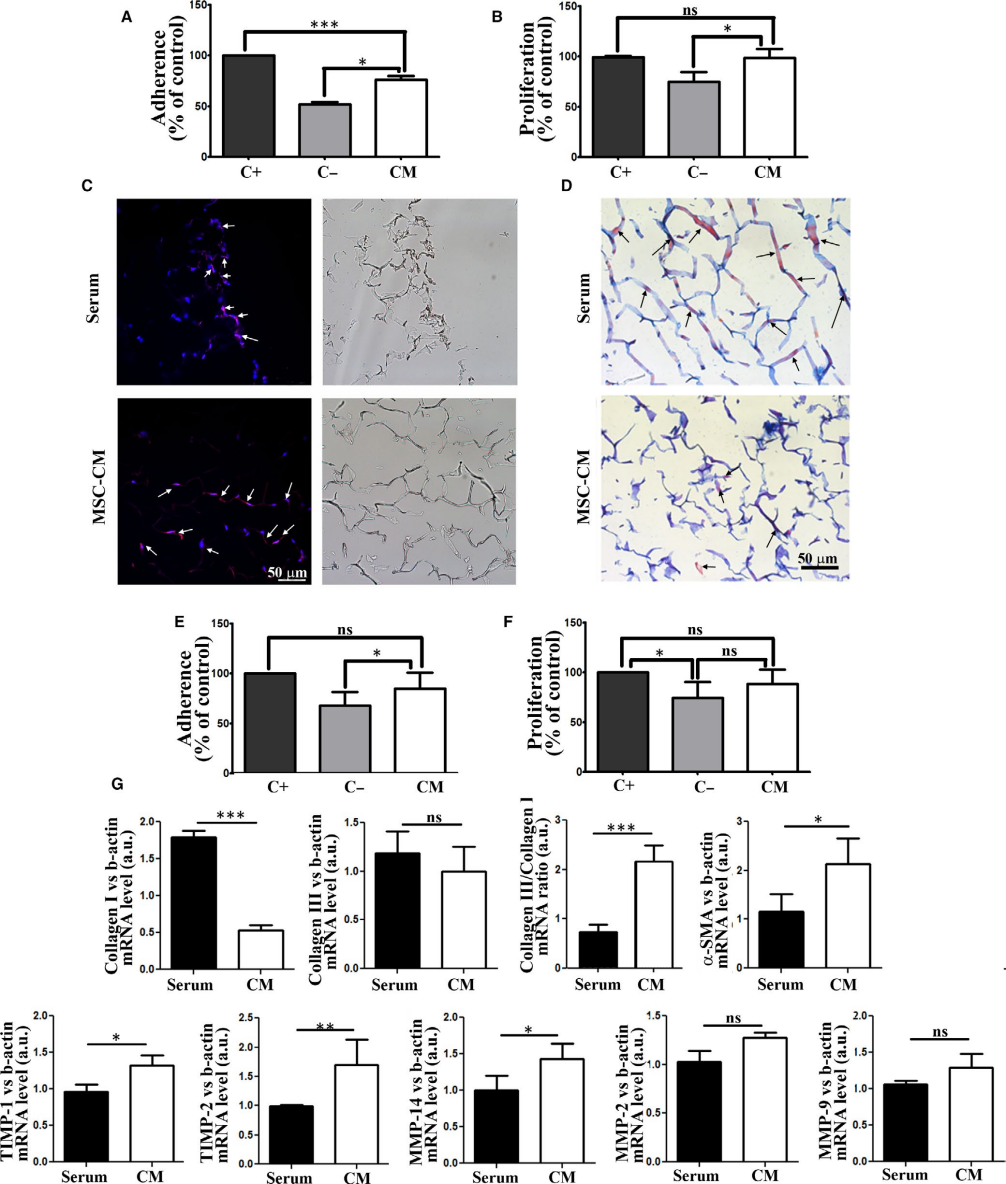
Assessment of MSC‐CM effect on keratinocytes and fibroblasts colonization of the collagen type I scaffold: A, Evaluation of the capacity of MSC‐CM to support keratinocytes adherence on 3D collagen scaffold, 24 hours after seeding; B, Assessment of the capacity of MSC‐CM to support of keratinocytes proliferation on 3D collagen scaffolds, after 5 days in culture; C, DAPI‐eosin staining and D, Ayoub‐Shklar staining illustrating the colonization of collagen 3D scaffolds with keratinocytes. The presence of cells attached to the collagen fibres are indicated by arrows; E, Evaluation of the capacity of MSC‐CM to support the adherence of dermal fibroblasts on 3D collagen scaffold, 24 hours after seeding; F, Assessment of the capacity of MSC‐CM to support the proliferation of dermal fibroblasts on 3D collagen scaffolds, after 5 days in culture; G, Real Time PCR showing the modification of fibroblast's gene expression for collagen I and II, α‐SMA, TIMP‐1, TIMP‐2, MMP‐14, MMP‐2 and MMP‐9 in the presence of MSC‐CM versus serum, when cultured on 3D collagen scaffolds

Given the encouraging effect of MSC‐CM on skin cells in classical 2D culture system, we subsequently assessed the effect of MSC‐CM on the colonization of the collagen scaffold. As shown in Figure 4A, MSC‐CM supports the adherence of keratinocytes to the scaffold (75 ± 4.2%) close to that obtained for the serum positive control (100%). Moreover, the proliferation of keratinocytes cultured on 3D scaffolds in the presence of MSC‐CM was similar to that found in the positive control (98.4 ± 8.8% of positive control), indicating a strong mitogenic effect (Figure 4B). The deep colonization of the scaffold with keratinocytes in the presence of MSC‐CM was shown by DAPI‐eosin (Figure 4C) and Ayoub‐Shklar histological staining (Figure 4D), which revealed the presence of these cells attached onto the collagen fibres, both on the surface as well as inside the structure.

As for the adherence of dermal fibroblasts, as shown in Figure 4E, MSC‐CM sustained this process (91.5 ± 10.5% of positive control), similar to the results obtained in 2D culture system. Regarding the proliferative effect, the value for MSC‐CM incubated cells was 88.4 ± 14.4% and 74.6 ± 15.8% for negative control, without a statistically relevance (Figure 4F), indicating only a mild mitogenic effect on fibroblasts.

During histological processing, we noticed that the fibroblasts colonized scaffolds seemed more damaged, especially for MSC‐CM samples (Figure S2). Therefore, we investigated the effect of MSC secretome on the ability of fibroblasts to contribute to the extracellular matrix remodelling. The Real Time PCR results indicated that MSC‐CM strongly impacted the gene expression of fibroblasts for collagen I and III, metalloproteinases (MMPs) and their inhibitors (TIMPs), both in 2D and 3D culture system. Thus, although the transcription of both collagen I and collagen III mRNAs are induced in 2D culture by MSC‐CM (2‐fold and 3‐fold higher, respectively, compared to serum control), as shown in Figure S3, the opposite mechanism was observed on collagen scaffolds (Figure 4G). However, the ratio between collagen III and I mRNAs was 2 to 3 times higher for the fibroblasts incubated in MSC‐CM, both in 2D and 3D systems.

Subsequently, we evaluated the effect of MSC‐CM on the expression of α‐SMA, an early marker of fibroblast differentiation towards myofibroblast. It was previously shown that, when cultured on plastic, fibroblasts gain a proto‐myofibroblast phenotype, including the expression of α‐SMA.^19^ As revealed by immunocytochemistry (Figure S4), our cells expressed α‐SMA in standard culture conditions, without organization in stress fibres. The PCR data indicated that while MSC‐CM did not have an impact on α‐SMA gene expression in 2D culture in comparison to serum, when cultured on type I collagen scaffold, the gene expression of α‐SMA was 2‐fold higher in the presence of MSC‐CM.

Regarding the gene expression of MMPs and TIMPS for the fibroblasts cultured on 3D collagen scaffolds in the presence of MSC‐CM, both the expression of TIMPs (1 and 2) and MMP‐14 was increased, while the expression of MMP‐2 and MMP‐9 was not significantly modified compared to the serum control (Figure 4G). In classical 2D system, MSC‐CM was able to up‐regulate the gene expression of TIMP‐1 and 2, MMP 14 and also MMPs 2 and 9 (Figure S3). We concluded that in the case of fibroblasts grown on collagen scaffold, MSC‐CM made these cells prone to participate in the wound remodelling phase, by presenting high collagen III versus collagen I ratio and a 2‐fold elevated expression of α‐SMA, TIMPs and MMP‐14, the latter being responsible for the degradation of fibrillar collagen.^20^

### 3.5 MSC‐CM has pro‐angiogenic properties

**FIGURE 5:**
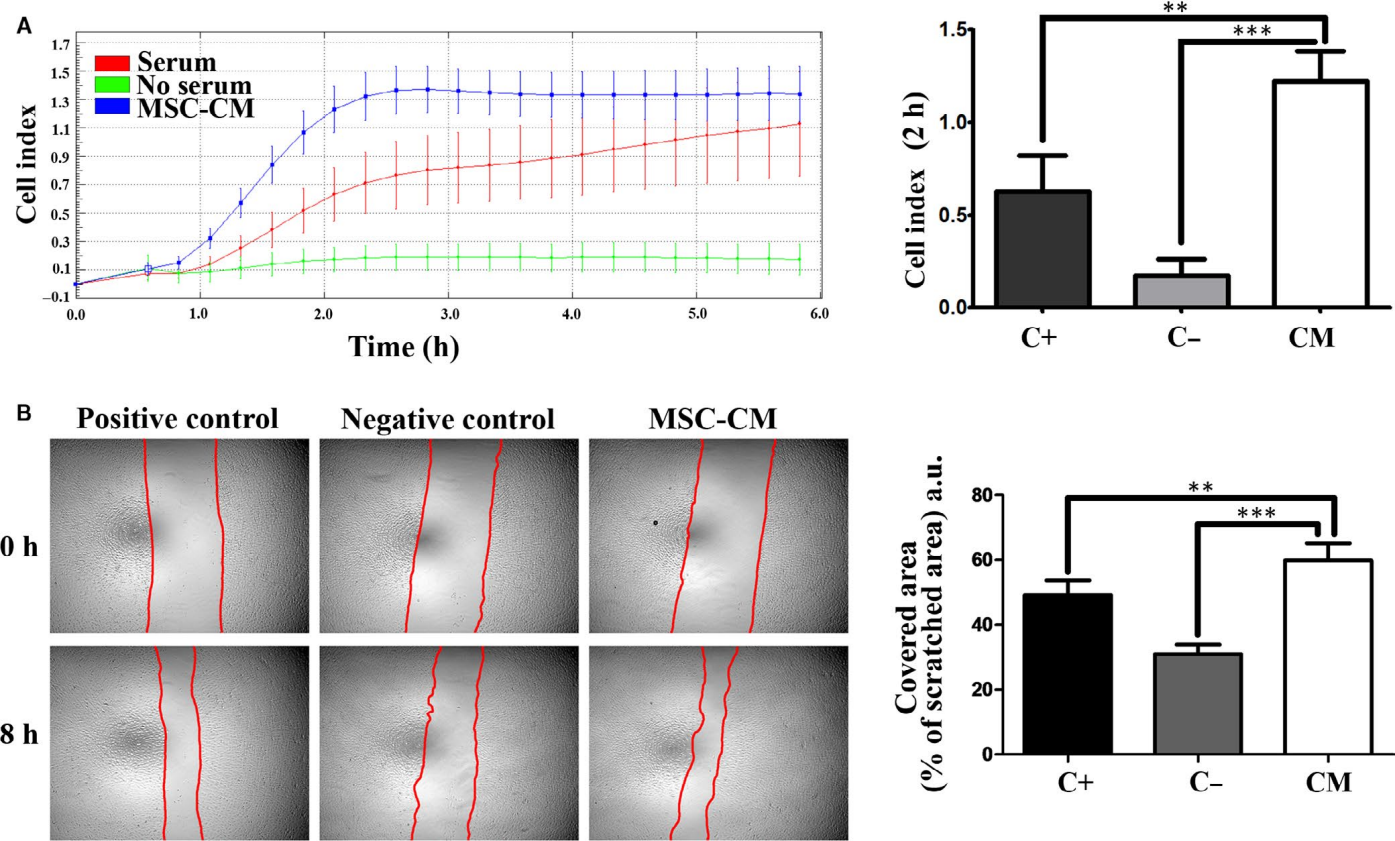
MSC‐CM has pro‐angiogenic effect on human endothelial cells. A, Time‐dependent assessment of chemotactic migration of endothelial cells. The diagram on the right illustrates the migration index at 2 h, when the chemotactic effect of MSC‐CM on endothelial cells was higher compared to the serum positive control; B, Scratch test assay on endothelial cells migration: (left) phase‐contrast microscopy showing the scratched area at t = 0 h and 8 h later and (right) the quantification of covered area as percentage of the initial scratched area

The importance of the angiogenic process in the wound healing mechanism is well established,^21^ and the pro‐angiogenic effect of MSC‐released factors is already documented for several tissues.^22^, ^23^ In the context of studying the MSC‐CM as adjuvant component in wound healing therapy and to corroborate this property with the results obtained on keratinocytes and fibroblasts, we assessed its effect on endothelial cells. The time‐dependent profile of chemotactic migration of endothelial cells in response to MSC‐CM showed a stronger chemoattractive effect reflected by a higher cell index compared to the positive control (serum) as shown in Figure 5A. Moreover, MSC‐CM stimulated endothelial cells migration as revealed by the scratch test, by inducing a coverage of the scratched surface area of 56.4 ± 7.2% versus 49.1 ± 4.5% in the positive control and 31 ± 3% in the negative control settings (Figure 5B). Our data showed that MSC‐CM had a pro‐angiogenic effect on human endothelial cells.

### 3.6 Evaluation of MSC‐secreted factors

**FIGURE 6:**
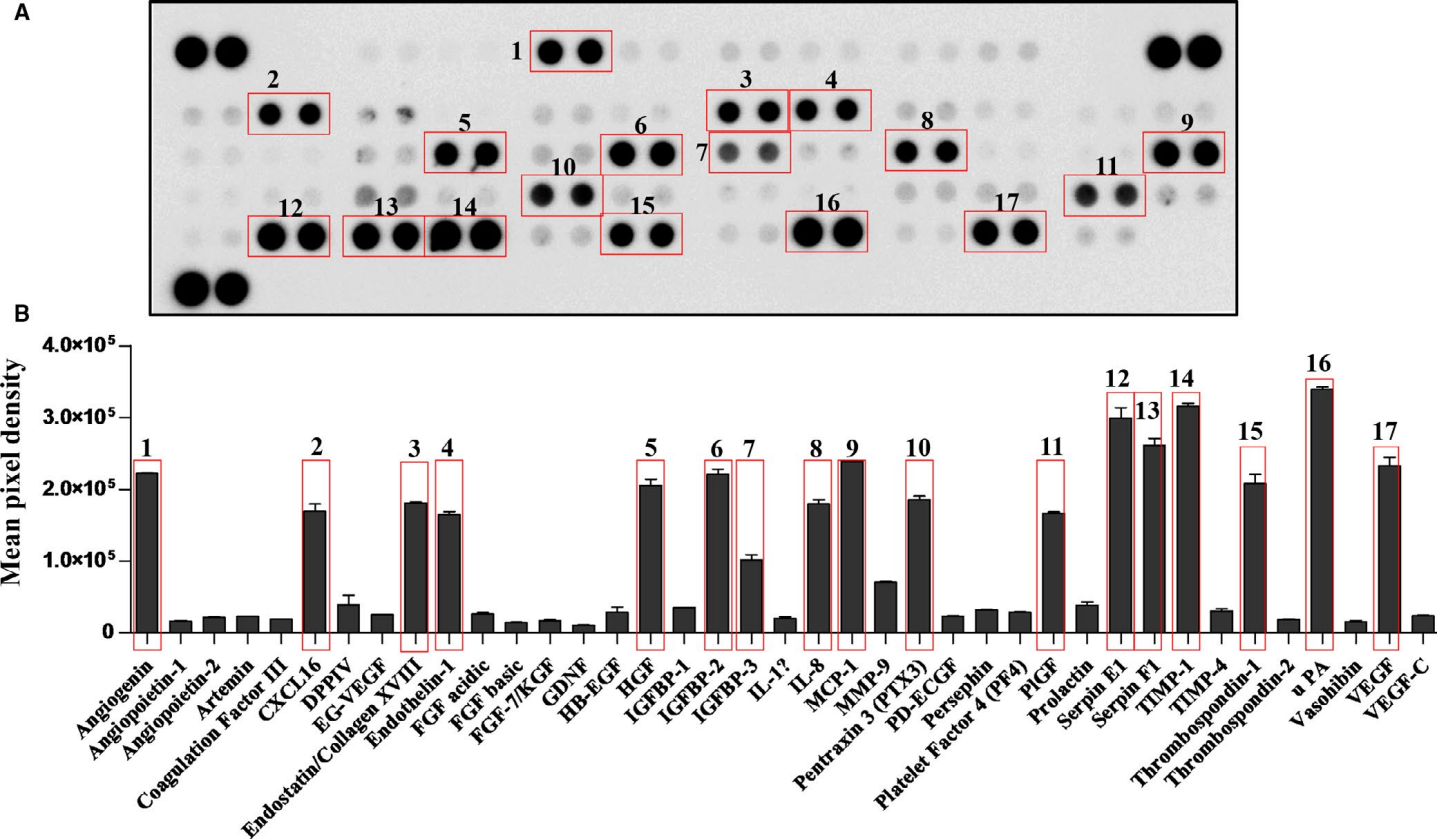
Evaluation of MSC‐CM composition: A, Cytokine array showing the composition of MSC‐CM regarding the pro‐ and anti‐angiogenic factors. The rectangles indicate the most abundant cytokines secreted by MSC; B, diagram depicting the relative quantification of the array

The secretory phenotype of MSCs was evaluated using a cytokine array (Figure 6A), which provided insight on the growth factors implicated in the effect of MSC‐CM on the proliferation of keratinocytes and fibroblasts. Thus, the growth factors traditionally implicated in the proliferation of keratinocytes, EGF and KGF, had low levels in MSC secretome (Figure 6B). Therefore, the proliferative effect on keratinocytes could be explained by the presence of HGF, which has been shown to have mitogenic properties and to stimulate keratinocytes motility.^24^, ^25^, ^26^ Furthermore, MSCs secreted only low levels of FGFs, which explained the lack of impact on fibroblast proliferation. Additionally, MSCs released factors with either pro‐ (angiogenin, CXCL16, endothelin‐1, HGF, IGFBP‐2 and 3, IL‐8, MCP‐1, PIGF, uPA, VEGF‐A) or anti‐angiogenic activity (endostatin, pentraxin 3, serpin E1, serpin F1, TIMP‐1, thrombospondin‐1), suggesting that MSC‐CM could be involved in the modulation of the angiogenic process by maintaining a balance between these factors.

## 4 DISCUSSION

The ideal therapeutic approach for chronic wounds is a cost‐effective, ‘off the shelf’ and topically applied biomaterial, which provides mechanical strength and delivers the suitable factors to promote wound healing.^27^ Recent in vitro engineered skin substitutes have various limitations such as expensive costs, abnormal skin microstructure and engraftment failure.^28^ Moreover, similar clinical outcomes have been reported for both acellular and cellular skin substitutes.^29^ A recent completed clinical trial comparing a cellular bioengineered substitute like Dermagraft and an acellular one‐ Oasis against standard care for patients suffering of diabetic foot ulcer, showed a complete healing for 78% of the patients who received the Oasis product compared to 57% for the ones who got the Dermagraft NCT01450943. Thus, it can be postulated that acellular scaffolds represent more promising tools for difficult to treat skin wounds compared to the more complex cellular scaffolds which are associated with a higher cost and shorter shelf life (days).

Therefore, we investigated the therapeutic potential of a porous scaffold made of type I collagen improved by the addition of a powerful trophic agent in the form of MSC‐CM in order to be used as an acellular scaffold for skin wound healing treatment. We chose to develop a type I collagen scaffold, as this is the most abundant and most readily available form of collagen, influencing various cellular properties and functions of fibroblasts and keratinocytes, including cell shape, adhesion, differentiation and migration 31,.^30^

As they were first described by Friedenstein and collaborators,^31^ MSC have been proposed as main candidates for regenerative therapy. Although they possess a certain differentiation capacity, recent insights in the mechanism by which these cells contribute to tissue regeneration indicate that their most important feature is the abundant secretion of bioactive trophic factors,^32^ which can modulate the local immune system, promote angiogenesis, prevent cell apoptosis, and stimulate survival, proliferation and differentiation of resident cells.^33^, ^34^, ^35^

The approach of combining the properties of a biomaterial and MSC‐CM in order to obtain a boosted therapeutic response has been successfully employed in several experimental settings, mainly for hard tissue regeneration. Thus, Osugi and collaborators showed the application of an agarose gel with MSC‐CM for calvarial defects had a superior regenerative effect compared to agarose gel mixed with MSC.^36^ Similar outcome has also been reported for collagen scaffolds supplemented with CM for the same type of tissue application.^37^ Recently, it was shown that a 3D structure containing carbon nanofibres functionalized with MSC‐CM improved cell adhesion, proliferation and viability and provided a biomimetic stem cell niche for cartilage and bone regeneration.^38^ Diomede and collaborators showed that a synthetic poly‐(lactide) scaffold enriched with human gingival MSC‐CM had an increased osteogenic action in a rat model of cranial defect.^39^

To our knowledge, the present paper is the first to explore this combinatorial approach involving a collagen scaffold and MSC‐CM for skin wound healing therapy. Here, we demonstrate that a natural‐derived biomaterial, such as the type I collagen scaffold combined with MSC‐CM has beneficial effects valuable in skin lesions treatment. Our data show that the secretome of bone marrow‐derived mesenchymal stromal cells: (i) promotes the colonization of the collagen scaffold with keratinocytes and fibroblasts, (ii) modifies the gene expression of molecules implicated in the synthesis and degradation of the extracellular matrix and (iii) has pro‐angiogenic properties. An interesting observation was the modification of the gene expression of dermal fibroblasts grown on the collagen scaffold: more exactly, the increase of the collagen III and I mRNAs ratio, of α‐SMA and TIMP‐1, TIMP‐2 and MMP‐14, but not MMP‐2 and MMP‐9 expression for the cells incubated in MSC‐CM compared to serum control. This effect could be particularly important in diabetic chronic wounds, where the fibroblasts are dysfunctional: the collagen production is decreased, MMP‐9 synthesis is increased and the overall balance between MMPs and TIMPs is disturbed, leading to the destruction of the extracellular matrix.^40^, ^41^

These data indicate that MSC‐CM induces a pro‐healing phenotype in fibroblasts, contributing to the contraction of the wound which requires α‐SMA activation, to the formation of the granulation tissue by increased collagen III/I ratio and to the biodegradation of the scaffold under the action of MMP‐14.

In conclusion, our data show that supplementing the collagen scaffold with MSC‐derived secretome results in an improved biomaterial, with enhanced therapeutic properties, such as boosted chemoattraction of epithelial and endothelial cells, while also contributing to the re‐epithelialization, matrix remodelling and angiogenesis processes. We stipulate that our approach, having the advantage of being an ‘off the shelf product’, as both the scaffold and the CM can be obtained in advance and stored until needed, could become an efficient therapeutic alternative for difficult to treat skin wounds.

## DISCLOSURES

The authors declare no conflict of interests.

## AUTHOR CONTRIBUTION

**Raluca Tutuianu:** Conceptualization (equal); Data curation (equal); Formal analysis (equal); Investigation (equal); Methodology (equal); Writing‐original draft (equal). **Ana‐Maria Rosca:** Conceptualization (equal); Data curation (equal); Formal analysis (equal); Investigation (equal); Methodology (equal); Writing‐original draft (equal). **Madalina Georgiana Albu Kaya:** Formal analysis (supporting); Methodology (supporting). **Vasile Pruna:** Formal analysis (supporting); Investigation (supporting). **Tiberiu Paul Neagu:** Resources (supporting). **Ioan Lascar:** Funding acquisition (supporting); Resources (supporting). **Maya Simionescu:** Supervision (supporting); Writing‐review & editing (supporting). **Irina Titorencu:** Conceptualization (lead); Funding acquisition (lead); Project administration (lead); Supervision (lead); Writing‐review & editing (lead).

## Supporting information

Supplementary Material

## Data Availability

The data that support the findings of this study are available from the corresponding author upon reasonable request.
