# Supplementary material for: Mesenchymal stromal cell‐derived factors promote the colonization of collagen 3D scaffolds with human skin cells
Source: J Cell Mol Med. 2020 Jul 14;24(17):9692–704. doi: 10.1111/jcmm.15507 (PMC7520263; doi:10.1111/jcmm.15507)
Supplement: Supplementary file 1 — Supplementary Material [file JCMM-24-9692-s001.doc]

**Table A.1**

| **gene** | **Primer sequence** |
| --- | --- |
| **Collagen I** | Forward: 5’- GTCACCCACCGACCAAGAAACC -3’  Reverse: 5’- AAGTCCAGGCTGTCCAGGGATG -3’ |
| **Collagen III** | Forward: 5’- GGTGTCCCAGGGAAAGATGG -3’  Reverse: 5’- TATACCTGGAAGTCCGGGGG -3’ |
| **α- smooth muscle actin** | Forward: 5’- GGCAAGTGATCACCATCGGA -3’  Reverse: 5’- GTGGTTTCATGGATGCCAGC -3’ |
| **TIMP-1** | Forward: 5’- ACTGATGGTGGGTGGATGAG -3’  Reverse: 5’- ATGGTGGGTTCTCTGGTGTC -3’ |
| **TIMP-2** | Forward: 5’- TGAGAAGGAAGTGGACTCTGG -3’  Reverse: 5’- CCTTTCCTGCAATGAGATATTCC -3’ |
| **MMP-2** | Forward: 5’- ACTACAACTTCTTCCCTCGCA -3’  Reverse: 5’- GGCATCATCCACTGTCTCTG -3’ |
| **MMP-9** | Forward: 5’- GCCACTACTGTGCCTTTGAG -3’  Reverse: 5’- CAGAGAATCGCCAGTACTTCC -3’ |
| **MMP-14** | Forward: 5’- TGAGGATCTGAATGGAAATGAC -3’  Reverse: 5’- GGGTTTATCAGGAACAGAAGG -3’ |
| **β-actin** | Forward: 5’- ACTCTTCCAGCCTTCCTTC -3’  Reverse: 5’- GATGTCCACGTCACACTTC -3’ |


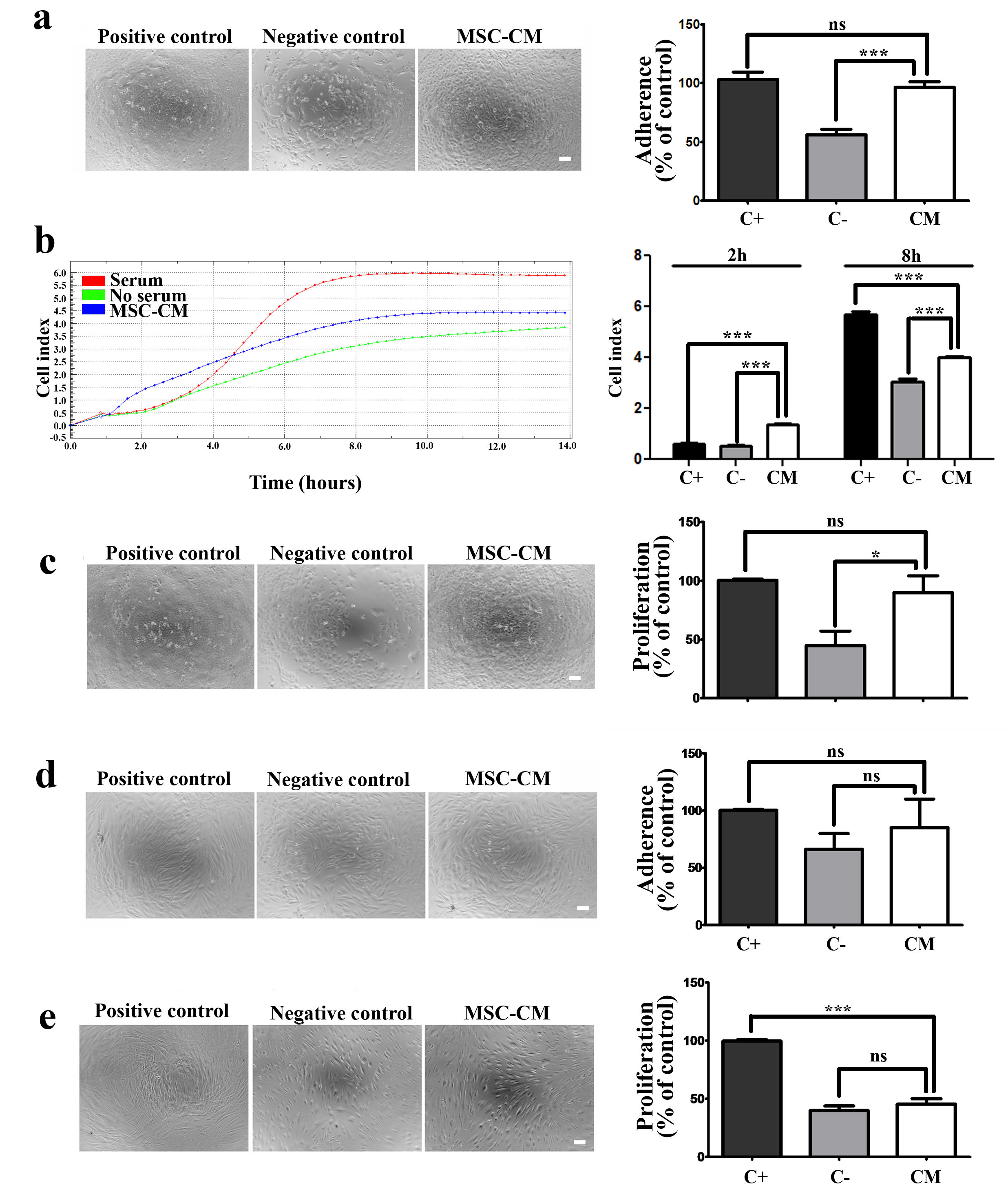


**Supporting figure A.1. Evaluation of MSC-CM effect on the adherence and proliferation of keratinocytes and fibroblasts in 2D culture system:** a) Assessment of the capacity of MSC-CM to promote keratinocytes adherence in 2D culture system: (left) phase-contrast microscopy showing the aspect of keratinocytes and (right) diagram representing the quantification of keratinocytes adherence 24h after seeding in the presence of MSC-CM versus serum the positive and negative controls; b) Representative diagram of real time assessment of the keratinocyte’s attachment to E-plate surface. Note that the MSC-CM induces keratinocytes attachment at a time earlier (2h) than the serum positive control (8h). The diagram (right) shows the quantification of the normalized cell index at 2 versus 8h, illustrating the difference between the dynamics of the adherence process sustained by MSC-CM versus positive and negative controls. The experiment was performed in quadruplicates; c) Assessment of the capacity of MSC-CM to promote keratinocytes proliferation in 2D culture system: (left) phase-contrast microscopy showing the aspect of the keratinocytes and (right) diagram representing the quantification of keratinocytes proliferation after 5 days in culture in the presence of MSC-CM versus serum the positive and negative controls; d) Evaluation of the capacity of MSC-CM to support the adherence of dermal fibroblasts in 2D culture system, 24h after seeding; (left) phase-contrast microscopy showing the aspect of dermal fibroblasts and (right) diagram representing the quantification of fibroblasts adherence 24h after seeding in the presence of MSC-CM versus serum the positive and negative controls; e) Assessment of the capacity of MSC-CM to support of the proliferation of dermal fibroblasts in 2D culture system, after 5 days in culture: (left) phase-contrast microscopy showing the aspect of fibroblasts and (right) diagram representing the quantification of dermal fibroblasts proliferation after 5 days in culture in the presence of MSC-CM versus serum the positive and negative controls.


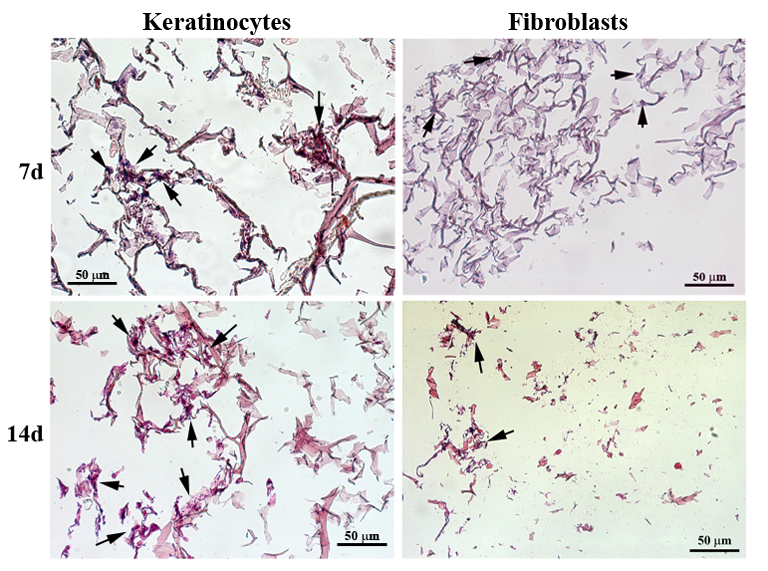


**Supporting figure A.2.** Histological analysis of collagen scaffold populated with human skin cells after one and two weeks in culture, respectively. The haematoxylin & eosin staining of collagen scaffolds cultured with keratinocytes and dermal fibroblasts for 1 and 2 weeks depicted a difference in the appearance of the sectioned collagen scaffolds. Thus, while the scaffolds cultured with keratinocytes appeared intact even after 2 weeks, the structure of those cultured with fibroblasts was heavily damaged over time, indicating the degradation of the collagen scaffold by the enzymes secreted by dermal fibroblasts.


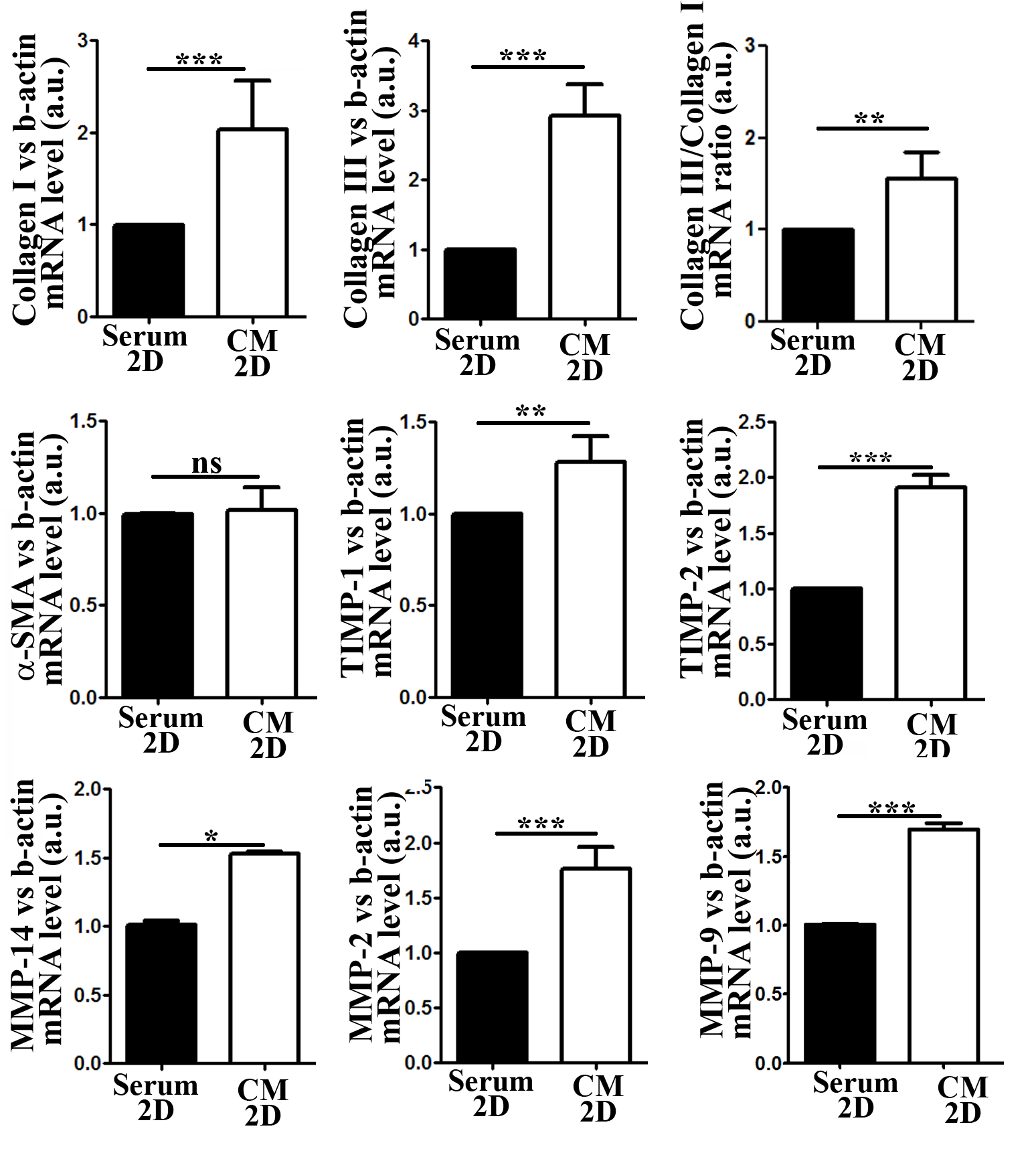


**Supporting figure A.3.** Real Time PCR showing the modification of dermal fibroblasts gene expression for collagen I and II, α-SMA, TIMP-1, TIMP-2, MMP-14, MMP-2 and MMP-9 in the presence of MSC-CM versus serum, when grown for 5 days in 2D classical culture system.


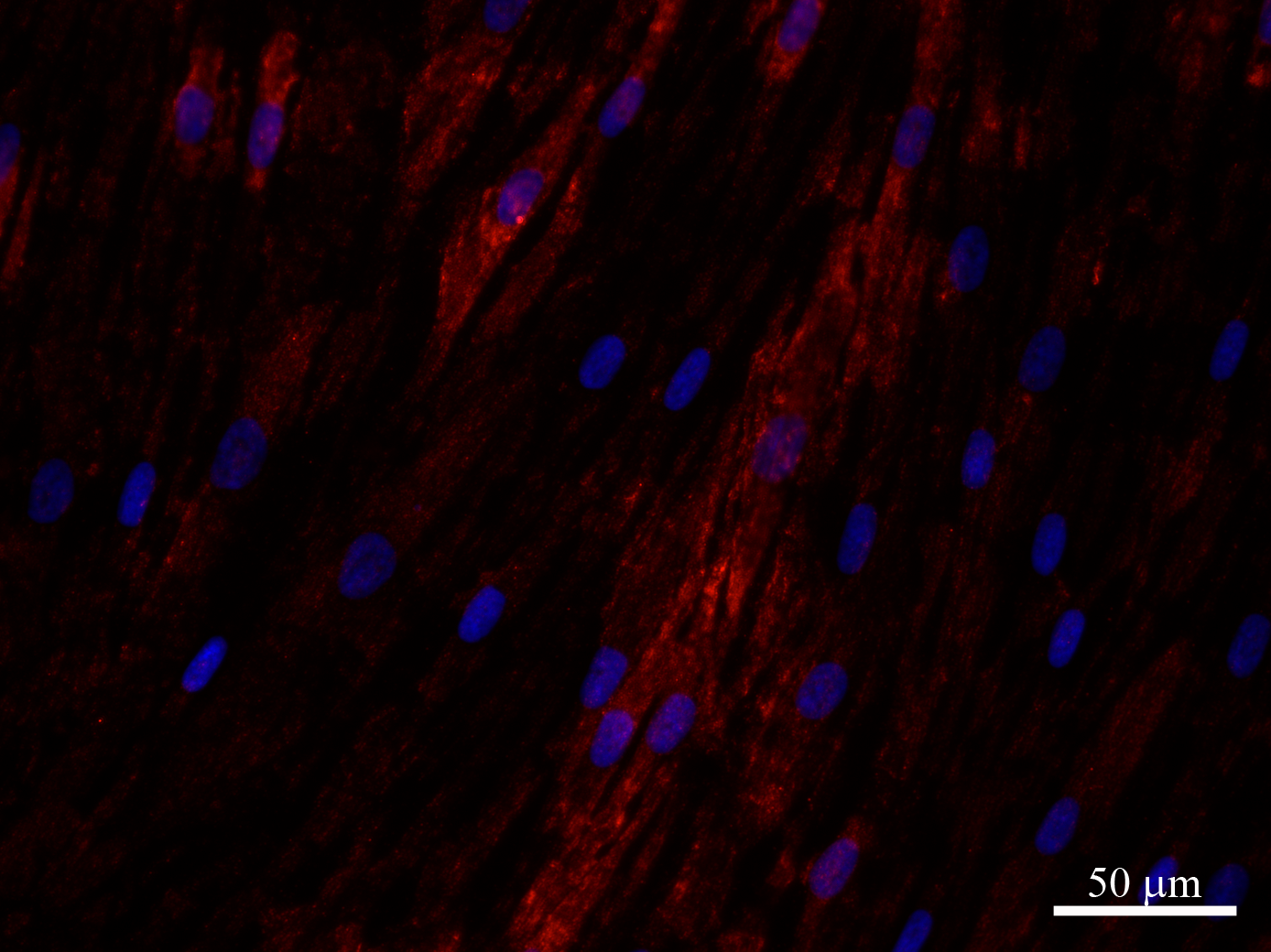


**Supporting figure A.4.** Immunocytochemistry image showing the expression of α-smooth muscle actin in human fibroblasts grown in classical 2D culture system.
